# Case Report: Diagnosis and treatment of an undifferentiated embryonal sarcoma of the liver

**DOI:** 10.3389/fsurg.2025.1481264

**Published:** 2025-04-23

**Authors:** Xiaojiao Qiu, Lexing Zhang, Fan Sun

**Affiliations:** ^1^ Department of Radiology, Yuhuan Second People’s Hospital, Yuhuan, China; ^2^ Department of Ultrasound, Hangzhou First People’s Hospital, Hangzhou, China

**Keywords:** undifferentiated embryonal sarcoma of the liver, hepatic sarcoma, neoplasm of the liver, surgery of the liver, case report

## Abstract

Undifferentiated embryonal sarcoma of the liver (UESL) is an exceedingly rare primary malignant tumor, predominantly affecting children and, to a lesser extent, adults. In adult patients, UESL constitutes a mere 7% of all liver sarcomas. This case report details an instance of hepatic embryonal sarcoma in a 33-year-old female patient who had no significant prior medical history and presented with complaints of abdominal pain. She was hospitalized for surgical intervention. Diagnostic imaging, including CT and MRI scans, disclosed a substantial cystic mass located in the right lobe of the liver. A lumpectomy was performed, and subsequent pathological and histological examinations confirmed the diagnosis of undifferentiated embryonal sarcoma. After surgery, the patient received adjuvant chemotherapy. We are pleased to report that she has remained in complete and sustained remission for the past 3 years.

## Introduction

Undifferentiated embryonal sarcoma of the liver (UESL), also referred to as malignant parenchymal tumor of the liver, is an exceedingly rare malignancy in adults, with fewer than 60 cases documented in the literature to date (1, 2). Predominantly affecting pediatric populations, this tumor typically emerges between the ages of 6 and 10 years and ranks as the third most frequent primary liver malignancy, following hepatoblastoma and hepatocellular carcinoma (3, 4). In the adult demographic, UESL accounts for a mere 7% of all liver sarcomas, with a notable predilection for women aged 40–50 years (5).

UESL is an aggressive tumor characterized by a tendency for local invasion and systemic metastasis. It often remains asymptomatic but can occasionally present with abdominal pain, which is typically the main symptom prompting patients to seek medical attention. Early diagnosis of this tumor offers a possibility for successful treatment and a favorable prognosis. Regrettably, due to the non-specific nature of clinical and imaging findings, misdiagnosis can occur, leading to delays in both surgical and systemic treatment. Surgery is generally the preferred initial treatment for UESL, with radical resection recommended for all cases.

In this report, we present the case of a middle-aged woman who experienced abdominal pain without any other distinct clinical signs. This patient underwent a radical tumor resection and was subsequently diagnosed with hepatic sarcoma.

## Case reports

### Clinical manifestations

**Figure 1:**
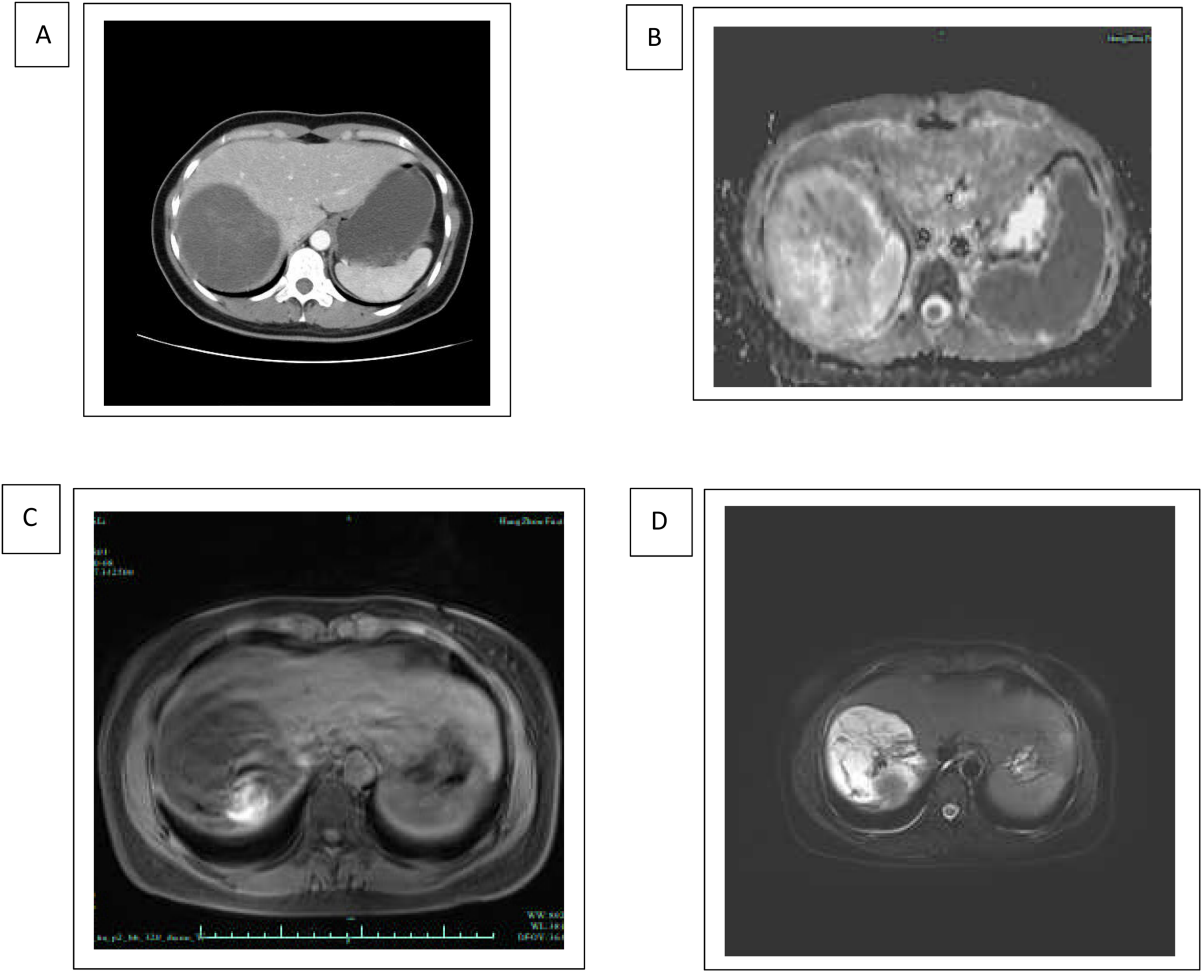
Radiology of undifferentiated embryonal sarcoma of the liver. **(A)** Axial contrast-enhanced CT scan of the upper abdomen demonstrating hepatomegaly with hypodense lesions in the liver. The hepatic lesion appears well-defined and hypodense, suggestive of possible tumor. **(B**–**D)** MRI of the upper abdomen demonstrating a hyperintense lesion in the liver, suggestive of a fluid-containing or highly vascular structure. The imaging characteristics of diffusion-weighted MRI **(B)**, T1-weighted MRI **(C)**, and T2-weighted MRI **(D)** are consistent with malignancy of embryonal sarcoma.

We hereby report the case of a 33-year-old female patient who was diagnosed with UESL. She presented at the hospital with a 2-day history of abdominal pain. Upon abdominal examination, no palpable mass was detected. Results from routine laboratory tests showed no signs of chronic liver disease and serum tumor markers were within normal limits. An ultrasound examination of the abdomen revealed a large cystic mass located in the right lobe of the liver. Further evaluation with computed tomography (CT) and magnetic resonance imaging (MRI) confirmed the presence of a 10 cm cystic mass in the same area. The CT images depicted a well-defined, low-density lesion without evident internal septations, which demonstrated progressive enhancement after the administration of a contrast agent. The MRI scans exhibited a low-intensity signal on T1-weighted images and a high-intensity signal on T2-weighted images, both of which showed enhancement after the injection of a contrast enhancer (Figure 1).

No abnormalities in liver function were detected, and serum tumor marker levels, including CEA, CA19.9, and alpha-fetoprotein, were within normal ranges. Viral markers also tested negative. After consultation with radiologists and oncologists, a decision was made to proceed with abdominal surgery.

Once general anesthesia was induced, the patient was positioned supine with the right side of the back elevated. A urinary catheter was inserted and the area was prepared with standard disinfection and draping. A roughly 30-cm L-shaped incision was made below the right costal margin to access the abdominal cavity, which was entered methodically, layer by layer.

During the exploratory phase of the surgery, a mass in the right posterior lobe of the liver was identified, firmly adherent to the diaphragm. No ascites or additional masses were observed within the abdominal cavity. The lesser omentum was incised and a hepatic pedicle clamp was applied. The round ligament and falciform ligament were divided and the second hepatic hilum was dissected. The right hepatic renal ligament was severed to expose the right side of the inferior vena cava (IVC). Two hepatic short veins were ligated and transected.

The coronary ligaments on both sides were mobilized and the tumor was detached from the diaphragm. The tumor's surface exhibited a rupture in the liver parenchyma, accompanied by a few blood clots. The site of liver rupture was sutured in an intermittent fashion. Electrocautery was employed to outline the resection margin on the tumor's surface. Hepatic resection was performed using intermittent application of hepatic needles along the resection line, gently lifting the liver. Ultrasonic shears (cavitron ultrasonic surgical aspirator (CUSA)) were utilized to fragment the liver tissue along the resection line. Metal clips were used to secure and divide any encountered blood vessels. Several branches of the right hepatic vein, which were observed entering the tumor, were properly ligated and transected. The tumor was completely excised, with no evident bleeding or bile leakage at the resection site.

The abdominal cavity was meticulously irrigated and a drainage tube was placed in the hepatorenal recess. The abdomen was then closed in layers, ensuring that no surgical instruments or gauze were left behind. The drainage tube was then securely positioned.

The surgical specimen was presented to the family and submitted for routine pathological examination. It displayed well-defined margins, measuring 9.5 cm × 8 cm × 7 cm, with a yellow-red coloration, colloid cystic, and hemorrhagic regions. Microscopic examination revealed a tumor composed of spindle cells exhibiting significant atypia and visible mitotic divisions, some of which were atypical. Approximately 20% of the tumors contained periodic acid-Schiff stain (PAS)-positive multinucleated giant cells.

**Figure 2:**
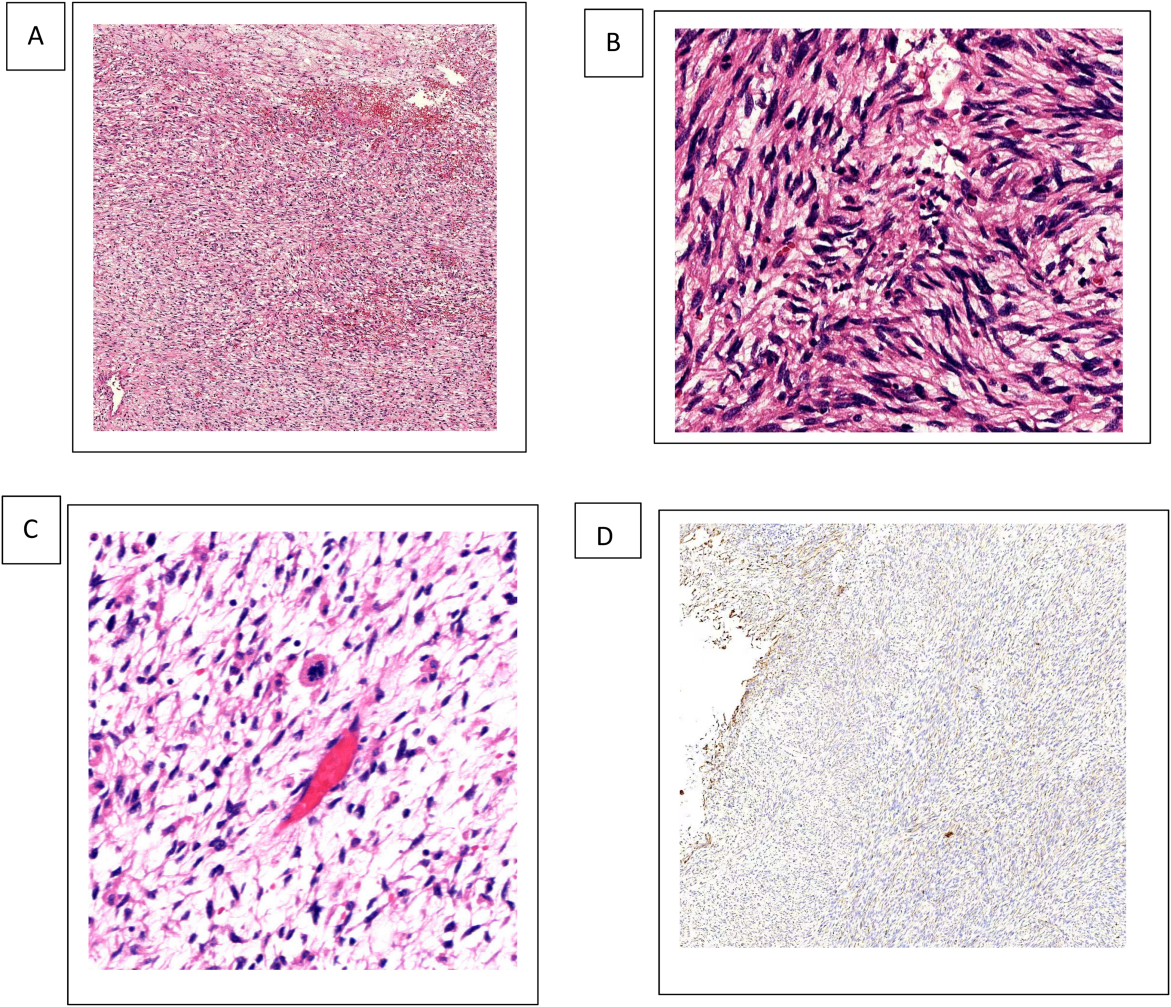
UESL tissue H&E staining and vimentin expression. **(A**–**C)** 4×, 10×, 20× H&E staining revealed spindle-shaped cells with ill-defined borders that make up the solid component of the tumor, giving it a sarcomatous appearance. **(B)** 4× IHC vimentin. IHC, immunohistochemistry.

Immunohistochemistry was carried out to help the diagnosis (Figure 2).

The tumor cells show diffuse vimentin positivity, as well as heterogeneous, focal, weakly positive desmin, alpha1-antitrypsin (α1-AT), and glypican-3. Smooth muscle actin, caldesmon, cluster of differentiation (CD)10, CD34, S100protein, and discovered on gastrointestinal stromal tumor-1 (DOG-1) staining are negative (6). In densely populated areas, ki67 staining is visible in approximately 30%–35% of cells. Other tumors that can be excluded and histologically matched tumor types are angiomyolipoma (melan A, S100 protein), gastrointestinal stromal tumor (DOG-1), epithelioid hemangioendothelioma (CD34), and angiosarcoma (CD34). Typically, leiomyosarcomas have at least two smooth muscle markers that are diffusely positive. In this case, smooth muscle actin or caldesmon were negative, two markers that have been reported to be positive in hepatic embryonal sarcoma (5).

After a clear histological diagnosis, we performed a multidisciplinary evaluation and the oncologist performed six-stage adjuvant therapy with epirubicin and cyclophosphamide, which the patient accepted. At 36 months postoperatively, the patient is alive with no recurrence.

## Discussion

The term “undifferentiated embryonal sarcoma” was first introduced by Stocker in 1978 to characterize a liver parenchymal tumor lacking specific differentiation (3). In the intervening 50 years, fewer than 60 cases have been documented, with patients’ ages in the range of 25–84 years, and a slightly elevated incidence among adult women, with no discernible difference in the pediatric population.

Typically, UESL presents without distinctive clinical signs, and radiological assessments often reveal a solid or cystic mass in the liver. There are no specific serum markers for UESL and imaging findings can vary. CT typically displays well-defined, low-density masses that are mostly cystic, frequently accompanied by internal septations and enhanced post-contrast injection.

UESL can occur anywhere in the liver but is most commonly found as a single, rapidly expanding mass in the right lobe. Symptoms are generally indicative of tumor growth and are characterized by pain and abdominal distension. No specific serum markers are diagnostic for this condition, although some studies have noted elevated liver enzymes and CA125 levels in certain cases.

Immunohistochemical analysis is crucial for confirming the diagnosis and differentiating UESL from other conditions. Histologically, UESL is marked by high-grade undifferentiated cells exhibiting a range of spindle and mucinous changes. To date, no specific immunophenotype for UESL has been identified. Tumor cells typically contain multiple, eosinophilic, PAS-positive granules of varying sizes within their cytoplasm. Immunostaining reveals positivity for vimentin, α1-antitrypsin, and focal, weak positivity for cytokeratin, which are sufficient for the diagnosis of UESL.

Achieving a preoperative diagnosis of UESL is challenging, and a definitive diagnosis is contingent upon pathological examination. If the diagnosis remains uncertain, immunohistochemistry and electron microscopy may be necessary. It is important to distinguish UESL from other liver tumors, including malignant fibrous histiocytoma, sarcomatoid hepatocellular carcinoma, and hepatic angiosarcoma. In this particular case, the patient's coexisting hepatitis B led to a preliminary misdiagnosis of hepatocellular carcinoma. Among benign cystic masses, the most common differential diagnoses include intrahepatic hamartomas, hepatic echinococcosis, and hepatic cysts.

Given the high malignant potential, rapid growth, and propensity for distant metastasis—most frequently to the lungs—surgical resection is the most effective treatment. As UESL often does not coexist with cirrhosis and is typically an encapsulated solitary tumor, surgical exploration is warranted, even if it involves multiple liver lobes, provided liver function remains normal. Tumor size should not be considered an absolute contraindication to surgery. For cases of postoperative recurrence or inoperable tumors, interventional therapy and radiotherapy may be employed initially to reduce the tumor size, followed by surgical resection if possible. The majority of untreated patients have a survival expectancy of less than 1 year. A combination of surgical resection and chemotherapy can enhance the 5-year survival rate for UESL patients to approximately 15%.

From this, it is evident that surgical resection is the preferred initial treatment and the integration of surgery with comprehensive postoperative care is pivotal in improving patient survival duration and rate.

## Data Availability

The original contributions presented in the study are included in the article/Supplementary Material, further inquiries can be directed to the corresponding author.
